# Incorporation of Temperature and Plastic Strain Effects into Local Approach to Fracture

**DOI:** 10.3390/ma14206224

**Published:** 2021-10-19

**Authors:** Sergiy Kotrechko, Vladislav Kozák, Oleksandra Zatsarna, Galyna Zimina, Nataliya Stetsenko, Ivo Dlouhý

**Affiliations:** 1 G. V. Kurdyumov Institute for Metal Physics, National Academy of Sciences of Ukraine 36, Academician Vernadsky Blvd., UA-0380 Kyiv, Ukraine; serkotr@gmail.com (S.K.); alexsandra.ku@gmail.com (O.Z.); zimingal28@gmail.com (G.Z.); 2 E. O. Paton Institute for Materials Science and Welding, National Technical University of Ukraine “Igor Sikorsky Kyiv Polytechnic Institute” 37, Peremohy Ave., UA-03056 Kyiv, Ukraine; 3 Institute of Physics of Materials, Academy of Sciences of the Czech Republic, Zizkova 22, 61669 Brno, Czech Republic; kozak@ipm.cz (V.K.); dlouhy@fme.vutbr.cz (I.D.); 4 Institute of Materials Science and Engineering, Brno University of Technology, Technicka 2, 61669 Brno, Czech Republic

**Keywords:** fracture toughness, ductile-to-brittle transition, local approach to fracture, ferritic steel

## Abstract

An unjustified simplification of the local quantitative criterion regarding cleavage nucleation is a key problem in the utilisation of the Local Approach to Fracture (LA), particularly to predict the fracture toughness within the ductile-to-brittle transition (DBT) region. The theoretical concept of the effect of both temperature and the plastic strain value on the crack nuclei (CN) generation rate in iron and ferritic steels is presented. It is shown how the plastic strain and temperature affect CN formation rate and, as a consequence, govern the shape of the temperature dependence of fracture toughness *K_Jc_* and its scatter limits. Within the framework of the microscopic model proposed, dependences of the CN bulk density on the plastic deformation value and temperature are predicted. Convenient approximation dependences for incorporating this effect into the LA are suggested. The experimental data of reactor pressure vessel steel and cast manganese steel demonstrate that the use of these dependences enables one to predict, with sufficient accuracy, the effect of temperature on the value of fracture toughness and its scatter limits over the DBT region. It is shown that accounting for both the temperature and strain dependence of CN bulk density gives rise to the invariance of parameters of the Weibull distribution to temperature.

## 1. Introduction

The Local Approach (LA) to fracture was introduced in the 1980s as a probabilistic method accounting for microstructural effects in the evaluation of global parameters. It aimed to solve key problems of fracture mechanics such as the prediction of the specimen geometry and statistical size effect on fracture toughness (transferability problem), as well as the effects of test temperature, operation temperature, and degradation of the material due to neutron irradiation, etc. The possibility of solving these complex problems was seen in the employment of statistical local criterion for initiating fracture in the vicinity of a crack, because this approach accounts for the key features of the mechanism initiating fracture at the micro-scale.

The Local Approach is based on the postulation that the probability of cleavage initiation within the vicinity of a crack/notch is described by the Weibull distribution. Accordingly, the parameters of this distribution are material constants that do not depend on its stress–strain state and temperature [1]. This approach describes the temperature dependence and the scatter limits of the fracture toughness $K_{IC}$. In this case, the temperature dependence of the value $K_{IC}$ is governed by the temperature dependence of the magnitude of yield strength. However, in the region of the ductile-to-brittle-transition, where there is a rapid increase in the fracture toughness against the background of a slight decrease in the yield strength, especially for high-strength steels, this approach underestimates the slope of the temperature dependence of the fracture toughness curve. In order to compensate for this shortcoming, one has to agree that shape parameter *m* and scale factor $\sigma_{u}$ are not constants; their values depend on the temperature [2,3,4,5,6,7,8]. Moreover, the values of these parameters depend on specimen geometry [2,9,10] and local plastic strain value [6]. All this contradicts the initial statements of the Local Approach. A number of studies have attempted to solve this problem [11,12,13,14,15,16]. These works showed the need to address two key issues, namely: (i) the need to incorporate the effect of temperature and the magnitude of local plastic strain on the CN bulk density ρ; (ii) the need to account for the value of the threshold stress $\sigma_{\mathrm{th}}$. As for the latter, in fact, this is a methodical problem. It consists of the development of a technique for the experimental determination of $\sigma_{\mathrm{th}}$. Therefore, a simplified method for determining $\sigma_{\mathrm{th}}$ for structural steels was proposed [17]. According to this technique, the value of $\sigma_{\mathrm{th}}$ is determined based on the results of uniaxial tensile tests of 5–6 notched cylindrical specimens with special geometries (maximum diameter is 5 mm; minimum notched diameter is 5.2 mm; notch radius is 2 mm) at the boiling point of liquid nitrogen (−196 °C).

Ruggieri and Jivkov et al. [15,16] tried to incorporate the effect of plastic strain and test temperature on the number of CN forming in the local plastic zone ahead of a macrocrack tip. This was achieved by revising the expression for the Weibull stress. The nature of this revision was to consider the effect of both temperature and equivalent plastic strain on the CN (“cleavage initiators”) number. This has been accomplished by introducing an empirical “thinning function” that makes it possible to predict “how many cleavage initiators must be generated relative to a reference case (for example all particles)” depending on temperature and the plastic strain value. The values of constants in this function were determined by a calibration procedure using experimental data. This enables one to predict, with high accuracy, the critical values of the $J_{IC}$ integral, both for low and high temperatures within the DBT region.

These studies have convincingly exhibited that the employment of LA within the DBT region requires the consideration of the temperature and deformation dependence of the CN bulk density. Here, in this contribution, the authors emphasised the need to explain the reasons for the significant effect of temperature on the number of formed “cleavage initiators”. As attempts to answer this question, one considers the research results presented in [17,18,19,20], where a physical version of the LA to cleavage fracture was proposed and evidenced. This version of LA does not employ any empirical dependences for the probability of both CN formation and instability. These functions are derived from a detailed analysis of the processes of CN formation and unstable equilibrium in a polycrystalline aggregate. This approach enabled us to ascertain the influence of both the metal structure and the conditions of its loading on the probability of fracture. However, it turned out to be quite sophisticated for engineering calculations. At the same time, this approach can be used as a tool to analyse the key effects that control the initiation of cleavage in the vicinity of the macrocrack, and in particular to analyse the effect of temperature and the magnitude of plastic deformation on the CN formation rate inside of the “process zone”.

The paper presents ideas about the physical nature of the effect of temperature and plastic strain value on the intensity of CN generation during the plastic deformation preceding cleavage. Physically substantiated dependences of CN density on temperature and the plastic strain value are obtained. To account for this effect within the LA, approximate dependences of CN density on temperature and the value of equivalent plastic strain are proposed. By the example of two structural steels, the suitability of using these dependences in predicting the value of fracture toughness within the DBT region is validated.

## 2. Theory

The inhomogeneity of microplastic deformation, which gives rise to plastic deformation incompatibility on grain and interphase boundaries, is a general reason for the CN formation in polycrystalline solids. A generalised model of CN formation in a polycrystalline aggregate was proposed [19,20].

The specific feature of this model is that it describes the formation and instability of the CN, accounting for both the main structural features of the polycrystal (inhomogeneous distribution of grain sizes and statistical distribution of their orientations) and for the action of microstresses generated by the incompatibility of microplastic deformations in polycrystal.

This model can be adopted for the prediction of the cleavage fracture in structural steels with ferritic basic microstructures arising from the expression for the bulk density ρ of CN formed at a given value of local plastic strain:

(1) $$\rho =2\cdot \rho_{cb}\;\int_{t_{C}}^{t_{r}}g(t)\;dt$$

where

(2) $$g(t)=\frac{1}{2\pi }\exp\left(\frac{t^{2}}{2}\right)$$

and $\rho_{cb}$ is the carbide particle density.

In this equation, *t* is the magnitude of normalised shear microstresses $\xi_{ns}$ acting in slip systems:

(3) $$t=\frac{\xi_{ns}}{\sqrt{D_{\xi_{ns}}}}$$

Here, $D_{\xi_{ns}}$ is the variance of shear microscopic stresses $\xi_{ns}$. Additionally $t_{C}$ is the critical value of normalised microstresses at which the CN are formed:

(4) $$t_{C}=\frac{1}{k_{\sigma }}\cdot \left[M+\frac{1}{\bar{\sigma }}\cdot \sqrt{\frac{\xi_{C}}{Cd_{\max}}}-\beta \cdot \sqrt{\frac{\bar{e}}{d}}\right]$$

where $\bar{\sigma }$ and $\bar{e}$ are the equivalent macroscopic stresses and strains; *d* and $d_{\max}$ are the average and maximum (with a given probability) ferritic grain sizes; $\xi_{C}$ is the critical stress of CN formation as a result of the carbide particle cleavage; *M* is the orientation factor (for α−Fe *M* = 0.36); $k_{\sigma }$*, C,* β are the coefficients (for ferritic steels $k_{\sigma }$ = 0.225; *C* = 0.0336 N/m; β ≈ 2.57 MPa m^0.5^ [19]. Furthermore, $t_{r}$ is the critical value of normalised microstresses in which there is relaxation of incompatibilities in intergranular boundaries:

(5) $$t_{r}=\frac{1}{k_{\sigma }}\cdot \left[M+\frac{1}{\bar{\sigma }}\left(\frac{\xi_{Y}}{m_{b}}\cdot \sqrt{\frac{r}{d_{\max}}}-\beta \cdot \sqrt{\frac{\bar{e}}{d}}\right)\right]$$

where $\xi_{Y}$ is the critical stress of the relaxation onset; $m_{b}$ is the orientation factor for relaxation slip systems in intergranular boundaries; *r* is the distance from the grain boundary to the origin of microstress relaxation.

In Equation (5), the expression $\beta \sqrt{\bar{e}/d}$ specifies the value of shear microstresses caused by the interaction of a grain of average orientation M with the surrounding matrix plastically deformed to strain $\bar{e}$. In polycrystals, these stresses arise as a result of the accumulation of microplastic strain incompatibilities at grain boundaries. The magnitude of the microplastic strain incompatibility changes nonmonotonically with an increase in macroplastic strain $\bar{e}$. At small macroplastic strains, it grows and then begins to decrease as a result of the predominance of relaxation processes in the near-boundary regions. A transition from growth to a decrease in these incompatibilities occurs at the critical strain $e_{C}$. At the macroscale, the value of $e_{C}$ can be estimated based on the dependence of the cleavage fracture stress of smooth (unnotched) specimens, $\sigma_{f}$, on the value of strain preceding fracture. When the strain $e_{C}$ is achieved, this stress $\sigma_{f}$ reaches its minimum value. For common structural steels with basic ferritic microstructures, $e_{C}$ ≈ 0.02 [10]. With an increase in ferrite grain size by annealing, the critical strain $e_{C}$ can increase to ≈ 0.05 [10]. Thus, dependences (4) and (5) are valid for strains not exceeding the critical one $e_{C}$.

**Figure 1:**
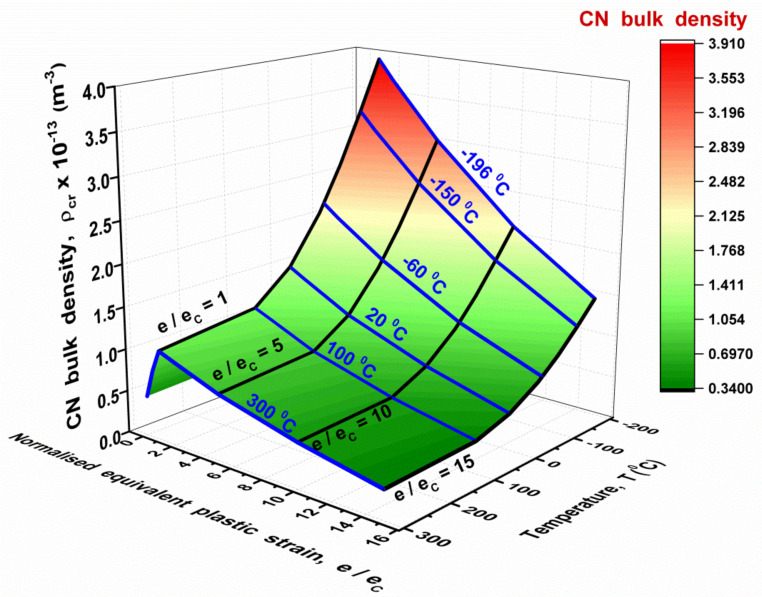
(Colour online). The effect of temperature *T* and plastic strain value $\bar{e}$ on the CN density ρ for the RPV steel: $\bar{e}$ is the equivalent plastic strain; $e_{C}$ is the critical value of strain ($e_{C}$ = 0.02).

At strains $\bar{e}$, greater than $e_{C}$, the expressions for $t_{C}$ and $t_{r}$ may be presented as follows:

(6) $$t_{C}=\frac{1}{k_{\sigma }}\cdot \left\{M+\frac{1}{\bar{\sigma }}\left[\sqrt{\frac{\xi_{C}}{Cd_{\max}}}-\beta \cdot \sqrt{\frac{e_{C}}{d}}+k_{e}\cdot \left(\frac{\bar{e}}{e_{C}}-1\right)\right]\right\}$$

where $k_{e}$ is the coefficient, characterising the intensity of relaxation process in the near-boundary regions. An estimate of this coefficient based on the [10] data (Figure 1) gives the value of $k_{e}$ ≈ 1.52 MPa m^0.5^.

Or, as the case may be, $t_{r}$:

(7) $$t_{r}=\frac{1}{k_{\sigma }}\cdot \left\{M+\frac{1}{\bar{\sigma }}\left[\frac{\xi_{Y}}{m_{b}}\cdot \sqrt{\frac{r}{d_{\max}}}-\beta \cdot \sqrt{\frac{e_{C}}{d}}+k_{e}\cdot \left(\frac{\bar{e}}{e_{C}}-1\right)\right]\right\}$$

The theoretical dependences of CN bulk density ρ on the magnitude of plastic strain and temperature for reactor pressure vessel (RPV) RPV steel are shown in Figure 1.

The following values of microscopic parameters were used in their building: *r* = 1 µm (typical distance from grain boundary to stress relaxation source; this value is commensurate with the substructure parameters); $m_{b}$ = 0.1 (the average value of orientation factor for the relaxation source); *d* = 10 µm and $d_{\max}$ = 30 µm (mean and maximum ferrite grain values); $\xi_{C}$ = 7 GPa ($\xi_{C}\approx 0.1G$ where G≈70 GPa is the iron carbide shear modulus) [21].

In the first approximation, the mean value of $\xi_{Y}$ may be estimated by the value of the thermally activated component of the yield strength $\xi_{Y}\approx \tau_{Y}$. The expression for $\tau_{Y}$ is the following [22]:

(8) $$\tau_{Y}=0.5C_{1}\exp\left[-\left(C_{2}-C_{3}\ln\dot{e}\right)T\right]$$

where $\dot{e}$ is the plastic strain rate (for quasi-static tension $\dot{e}$ = 10^−4^·s^−1^); *C*_1_, *C*_2_ and *C*_3_ are the constants, which values for typical ferritic steels are: *C*_1_ = 1033 MPa, *C*_2_ = 0.0068 K^−1^, *C*_3_ = 0.000415 K^−1^ [22].

The equivalent stress value $\bar{\sigma }$ was calculated according to equation

(9) $$\bar{\sigma }=\sigma_{0.2}\cdot {\left(\frac{\bar{e}}{0.002}\right)}^{n}$$

where *n* is the work hardening exponent (value *n* = 0.05 was used) and $\sigma_{0.2}$ is the yield strength:

(10) $$\sigma_{0.2}=\sigma_{a}+C_{1}\exp\left[-\left(C_{2}+C_{3}\ln\dot{e}\right)T\right]$$

where $\sigma_{a}$ is the athermal component of $\sigma_{0.2}$.

**Figure 2:**
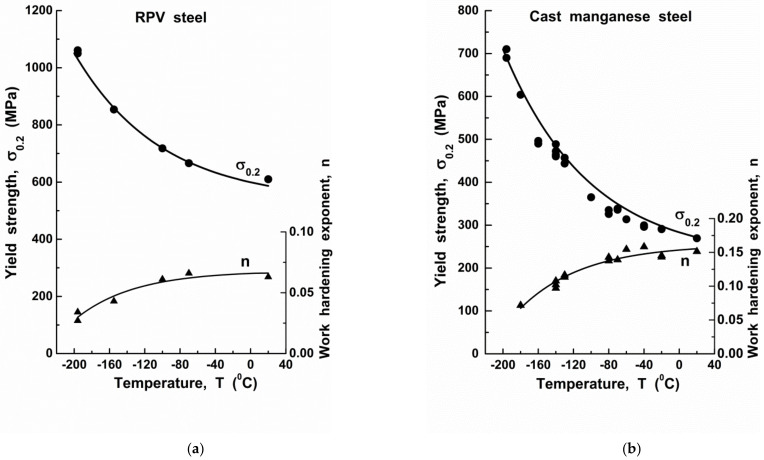
The temperature dependences of yield strength $\sigma_{0.2}$ and work hardening exponent n for RPV steel (**a**) and cast manganese steel (**b**).

For the investigated reactor pressure vessel (RPV) steel at room temperature (*T* = 293 K), the yield strength was measured to be $\sigma_{0.2}=610\;\mathrm{MPa}$. Accounting for Equation (10) and given the above *C*_1_, *C*_2_ and *C*_3_ values, the athermal component of the yield strength is $\sigma_{a}$ = 564 MPa. The temperature dependences of both yield strength $\sigma_{0.2}$ and the work hardening exponent n for RPV steel utilised in calculations are presented in Figure 2a. 

According to the calculation results (Figure 1), the non-monotonic dependence of ρ on the plastic strain value is a characteristic feature of the CN formation in polycrystalline metals. As was shown above, this is due to non-monotonic change in shear microstresses, induced by microplastic strain incompatibility at the grain boundaries, with macroplastic strain $\bar{e}$ growth. 

As the calculations show, the temperature increase should cause a monotonic decrease in the value of the CN density ρ. This is due to the thermally activated relaxation processes at the grain boundaries. In terms of the current model, this means a decrease in the value $t_{r}$ (dependences (7) and (5)) against the background of increasing the critical value of normalised stress of CN formation $t_{C}$ (dependences (6) and (4)).

Figure 1 shows that the effect of plastic strain magnitude and temperature on the CN bulk density ρ is a non-additive one. The maximum influence of plastic deformation on ρ is observed at strains close to the critical one $e_{C}$. With this deformation, the maximum sensitivity of ρ to temperature changes is also observed. At large strains, the sensitivity of ρ to changes in temperature decreases. In general, a comparison of the contribution of plastic strain and temperature to change in the intensity of the crack nuclei formation shows that the prevailing contribution is made by the change in temperature. An increase in temperature from +100 °C to −196 °C can lead to an almost fourfold increase in ρ. A decrease in temperature also increases the sensitivity of ρ to the plastic strain value. The dependences of CN density described above were obtained based on the analysis of the process of CN forming at microscale. Therefore, they enabled us to ascertain the fundamental regularities of the influence of temperature and plastic strain on the value of CN bulk density ρ, as well as determine the physical nature of this effect. However, these dependences *cannot be directly used* in LA since they contain microscopic parameters, the values of which can be estimated only with an accuracy of an order of magnitude. To incorporate these regularities into the LA, it is important to find the appropriate functions to approximate these dependences ρ on the temperature and the magnitude of plastic strain. The constants in these functions characterise the influence of microstructure parameters of specific steel on the sensitivity of ρ to both temperature and the magnitude of plastic strain. The values of these constants can be estimated from experimental evidence by means of calibration procedures.

In this case, as a first approximation, the following expressions can be used:

(11) $$\rho =\rho_{C}-a\cdot \left(1-\frac{\bar{e}}{e_{C}}\right)\;\mathrm{for}\;\bar{e}\leq e_{C}$$

and

(12) $$\rho =\rho_{C}-b\cdot \left(\frac{\bar{e}}{e_{C}}-1\right)\;\mathrm{for}\;e_{\max}\geq \bar{e}\geq e_{C}$$

where

(13) $$\rho_{C}=\rho_{0}\cdot \left[1-\exp\left(-\alpha \tau_{Y}\right)\right]$$

In the above equation, α and $\rho_{0}$ are coefficients, the values of which are calculated using experimental evidence by the calibration procedure, $e_{C}$ = 0.02 and $e_{\max}\leq 0.3÷0.5$.

The dependences in Figure 1 give the following values: *a* = 1.50 × 10^13^ m^−3^; *b* ≈ 0.12 × 10^13^ m^−3^. 

## 3. Experiment

**Figure 3:**
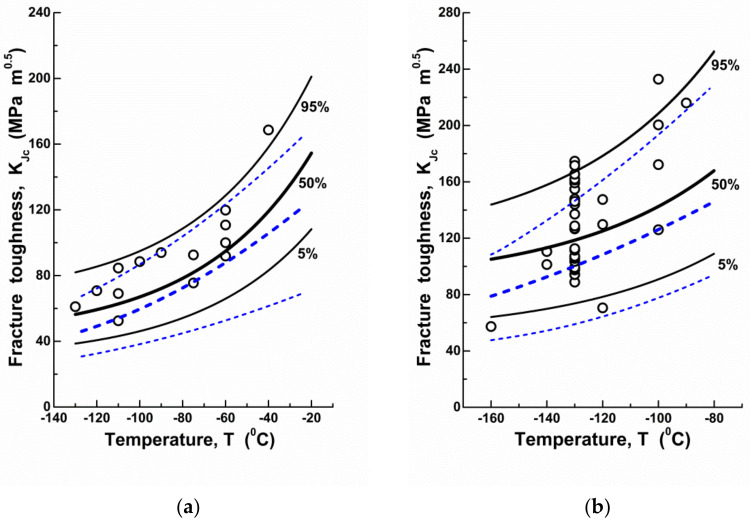
(Colour online). The temperature dependences of fracture toughness $K_{Jc}$ for RPV steel (**a**) and cast low-alloyed manganese steel (**b**): circles represent the experimental values; lines are the calculation results (solid black lines are the results of $K_{Jc}$ calculation accounting for temperature dependence for the CN density ρ, dashed blue lines are the same at the constant values of ρ).

**Figure 4:**
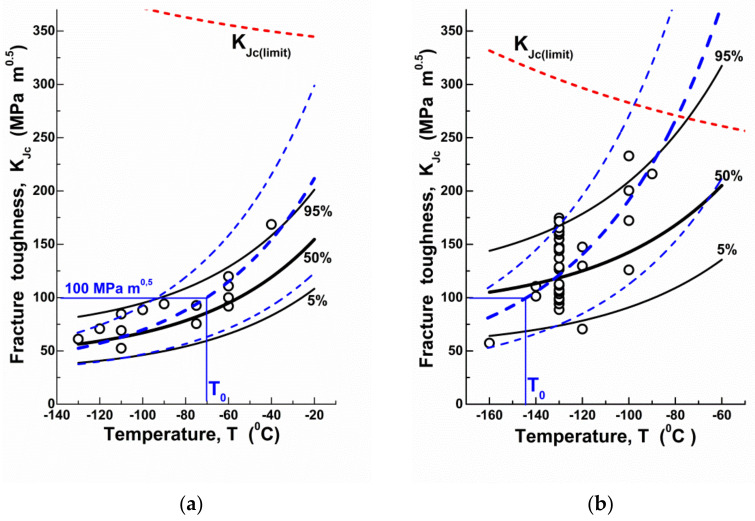
(Colour online). The temperature dependences of fracture toughness $K_{Jc}$ for RPV steel (**a**) and cast low-alloyed manganese steel (**b**): circles show the experimental evidence, lines are the calculation results (solid black lines are the results of $K_{Jc}$ calculation accounting for the CN density ρ and dashed blue lines are the results of $K_{Jc}$ calculation by the Master Curve method); $T_{0}$ is the reference temperature (short-dashed red lines are the temperature dependences of elastic plastic fracture toughness validity limit $K_{Jc\left(\mathrm{limit}\right)}$), calculated according to Equation (14).

Experimental investigations were performed on a reactor pressure vessel steel and on a low–alloyed manganese steel. To determine the value of fracture toughness, standard (ASTM E1921-05 [23]) specimens 1T CT (for the reactor pressure vessel steel) and 1T SENB (single edge notched bending) (for the low–alloyed manganese steel) were used. Chemical composition of the steels is presented in Table 1. More details on materials used can be found in our previous works, e.g., [17,24,25]. Both steels were investigated as received and had a ferritic structure. Mechanical tests of the pre-cracked specimens were executed in the DBT temperature range (Figure 3). The specimens were tested under crosshead speed of 1 mm/min in either environmental and/or cryogenic chamber. Experiments were carried out on a servo-hydraulic testing machine, Instron 8802. The value of $K_{Jc}$ was estimated according to the ASTM E1921-05 standard. According to this standard, the maximum limiting the valid values of elastic plastic fracture toughness, $K_{Jc\left(\mathrm{limit}\right)}$, was calculated according to the equation:

(14) $$K_{Jc\left(\mathrm{limit}\right)}=\sqrt{\frac{Eb_{0}\sigma_{0.2}}{30\left(1-\nu^{2}\right)}}$$

where *E* is the Young modulus, (*E* = 210 GPa); ν is Poisson’s ratio (ν = 0.27); $b_{0}$ is the unbroken ligament ($b_{0}$ = 22.4 mm); $\sigma_{0.2}$ is the yield strength (the temperature dependences for $\sigma_{0.2}$ of the investigated steels are presented in Figure 2). The temperature values of $K_{Jc\left(\mathrm{limit}\right)}$ are presented in Figure 4. To determine the mechanical properties of the investigated steels and obtain true stress vs. true deformation curves, low-temperature tests for uniaxial tension of smooth (unnotched) specimens were performed. According to the results of these tests, the values of the yield stress and the strain hardening exponent were determined (Figure 2).

According to the results of these tests, the magnitudes of Weibull parameters were determined by calibration (shape parameter *m* and scale parameter $\sigma_{u}$) as well as the values of $\rho_{0}$ and α. To determine local parameters, a program was created that allows the calculation of local parameters using the maximum likelihood method for a three-parameter modification of the Beremin approach. This is a typical procedure for the Local Approach. The difference is only that $V_{0}$ is not constant but depends on ρ ($V_{0}=\sqrt{1/\rho }$),the value of which is determined by dependences (11) and (12).

The magnitude of threshold stress $\sigma_{th}$ was estimated by the technique described in [17] (Table 2).

## 4. Results and Discussion

Relatively small *m* values (*m* ≤ 10) are the consequence of accounting for the values of threshold in the calculation [26]. During the calibration procedure, it was possible to find such a set of Weibull parameters, at which the values of $\rho_{0}$ and α for both steels are almost the same (Table 3). This validates the ascertained values $\rho_{0}$ and α as those inherent to ferritic steels. In this case, the features of the microstructure of steels are accounted for by the values of the Weibull parameters. It should be emphasised that accounting for the ρ temperature dependence ρ (in terms of the Weibull distribution—for the parameter $V_{0}$ ($\rho =V_{0}^{-1}$) gives rise to the independence (invariance) of the Weibull parameters $\sigma_{u}$ and *m* on temperature. This allows us to consider $\sigma_{u}$ and *m* as material parameters. Contrarily, in the conventional versions of Local Approach, it is assumed that the quantity $V_{0}$ is constant. This leads to temperature dependences of parameters $\sigma_{u}$ and *m,* which contradicts the physical sense and complicates calculations.

Based on these data, temperature dependences of fracture toughness $K_{Jc}$ for the studied steels were plotted (Figure 3a,b). The algorithm of calculations of fracture toughness and fracture probabilities was as follows:

1. For each *j^th^* stage of loading $K_{I}^{j}$, the values of equivalent plastic strain ${\bar{e}}^{i}$ and normal tensile stresses $\sigma_{YY}^{i}$ in each $i^{th}$ finite element were calculated using the finite elements method (software ABAQUS, version 2018).

2. Further, the probability of the cleavage initiation in the $i^{th}$ element was calculated as

(15) $$P_{i}=1-\exp\left[-\frac{V^{i}}{V_{0}^{i}}\times {\left(\frac{\sigma_{YY}^{i}-\sigma_{th}}{\sigma_{u}}\right)}^{m}\right]$$

where $V^{i}$ is the finite element volume and $V_{0}^{i}$ is the volume per one crack nucleus ($\rho =V_{0}^{-1}$). The value of $\rho^{i}$ was calculated according to Equations (11)–(13).

3. Then, the total probability of cleavage initiation in specimen is determined as

(16) $$P_{\Sigma }=1-\prod_{n=1}^{n=N}\left[1-P_{i}\left(n\right)\right]$$

where *N* is the number of finite elements belonging to the yield region.

The step size and the number of loading stages were chosen in such a way as to obtain $K_{I}$ values when the probabilities of cleavage initiation $P_{\Sigma }$ are 5%, 50% and 95%.

In accordance with the calculation results, the theoretical curve describes the temperature dependence of fracture toughness $K_{Jc}$ with a 90% probability scatter band (Figure 3). It should be noted that such a coincidence is due to the fact that not only a decrease in yield strength, but also a reduction in the CN generation rate is the reason for $K_{Jc}$ growth with increasing temperature. If we do not take into account this fact, i.e., if we assume that ρ = *const*, as the case may be, $V_{0}^{}$ = *const* ($V_{0}^{}$ = 1/ ρ), then the slope of $K_{Jc}$ temperature dependence becomes unbelievably low (Figure 3, dashed lines). A particularly significant error occurs at low values of the failure probability, i.e., when determining $K_{Jc}$ threshold values). This is especially important for applications. It should be mentioned that accounting for ρ temperature dependence provides invariance to temperature of the Weibull distribution parameters, which agrees well with the LA concept that considers *m* and $\sigma_{u}$ as material parameters.

The proposed approach can also be used to analyse the Master Curve method. For this purpose, the values of reference temperatures $T_{0}^{}$ were determined for the studied steels by the multitemperature method, and, according to the ASTM E1921-05 standard, the temperature dependences of fracture toughness were plotted (Figure 4).

According to these data, the Master Curve method overestimates the value $K_{Jc}$ at temperatures above the reference one $T_{0}^{}$. This means the need to “correct” the width of the temperature range near $T_{0}^{}$, within which the approximation of experimental evidence by the Master Curve method, is valid. The approach accounting for the temperature dependence of CN density formation may be used as a tool to fix this problem.

Fracture toughness is a stochastic characteristic. This is most pronounced within the DBT region. Therefore, it is necessary to pay attention to errors not only of $K_{Jc}$ absolute values, but also to errors of probabilities of their realisation. Figure 4 exhibits that the error in the $K_{Jc}$ value due to neglecting of ρ temperature dependence is in the tens of percent. At the same time, the error in probabilities of realisation of the given $K_{Jc}$ values can be quite high.

## 5. Conclusions

1. In a general case, the incompatibility of microplastic deformations at grain or interphase boundaries causes the crack nuclei (CN) formation. The value of this incompatibility depends on the magnitude of plastic strain and temperature. This is the reason for the influence of these factors on the intensity of the crack nuclei generation. The microscopic model presented in the work enables one to describe, quantitatively, the regularities of this effect.
2. The strain and temperature dependence of the CN bulk density results in the invariance of the Weibull distribution parameters both to the temperature and the plastic strain value, i.e., $\sigma_{u}$ and *m,* may be considered as material parameters. This is in line with the original concepts underlying the Local Approach.
3. Decreasing CN generation intensity with temperature growth is one of the key factors that governs the slope of $K_{Jc}$ temperature dependence and its scatter limits. Ignoring this effect underestimates the slope of $K_{Jc}$ temperature dependence and their absolute values. Especially large, up to 20–30%, is the error at low probabilities $P_{\Sigma }$ = 5% fracture (lower fracture toughness threshold).
4. The Master Curve method, vice versa, overestimates $K_{Jc}$ sensitivity to temperature. This may result in the overestimation of the fracture toughness value at temperatures higher than the reference one $T_{0}$.

## Figures and Tables

**Table 1:** Chemical composition of the investigated steels (wt %, remainder is *Fe*).

C	Mn	Si	Cr	Ni	Mo	Cu	S	P	V	As	Co	Sn	Sb
**RPV Steel**													
0.17	0.46	0.27	2.18	1.19	0.54	0.13	<0.01	0.01	0.10	<0.003	<0.03	0.006	<0.003
**Cast Ferritic Steel**													
0.09	1.18	0.37	0.12	0.29	0.03	0.29	0.025	0.01	N/A	N/A	N/A	N/A	N/A

**Table 2:** Mechanical properties of the tested steels ($\sigma_{0.2}$ is the yield strength determined as 0.2 pct proof stress; $\sigma_{ul}$ is the ultimate tensile strength; $e_{u}$ is the uniform elongation; ψ is the reduction in area—all at room temperature; *n* is the work hardening exponent).

Material	$\sigma_{0.2}(\mathrm{MPa})$	$\sigma_{ul}(\mathrm{MPa})$	$e_{u}$ (---)	ψ (---)	*n* (---)
RPV steel	610	714	0.07	0.75	0.06
Cast steel	319	481	0.26	0.74	0.15

**Table 3:** Calculation findings ($\sigma_{th}$ is the threshold stress; *m* and $\sigma_{u}$ are the shape and scale parameters; $\rho_{0}$ and α are the coefficients; $T_{0}$ is the reference temperature).

Material	$\sigma_{th}(\mathrm{MPa})$	$\sigma_{u}(\mathrm{MPa})$	*m*	$\rho_{0}\times \;10^{13}\;(m^{-3})$	α (MPa^−1^)	$T_{0}({}^{\circ}C)$
RPV steel	1100	6835	5.4	4	0.024	−70
Cast steel	720	3700	8.0	4	0.020	−145

## Data Availability

Data supporting the findings of this study are available from the corresponding author upon request.
